# Opportunities to Target Specific Contractile Abnormalities with Smooth Muscle Protein Kinase Inhibitors

**DOI:** 10.3390/ph3061739

**Published:** 2010-05-26

**Authors:** Annegret Ulke-Lemée, Justin A. MacDonald

**Affiliations:** Smooth Muscle Research Group and Department of Biochemistry & Molecular Biology, University of Calgary, 3280 Hospital Drive NW, Calgary, Alberta, T2N 4Z6, Canada; E-Mail: aulke@ucalgary.ca (A.U-L)

**Keywords:** calcium sensitization, zipper-interacting protein kinase, integrin-linked kinase, Rho-associated kinase, myosin phosphatase, myosin light chain diphosphorylation, MYPT1, CPI-17

## Abstract

Smooth muscle is a major component of most hollow organ systems (e.g., airways, vasculature, bladder and gut/gastrointestine); therefore, the coordinated regulation of contraction is a key property of smooth muscle. When smooth muscle functions normally, it contributes to general health and wellness, but its dysfunction is associated with morbidity and mortality. Rho-associated protein kinase (ROCK) is central to calcium-independent, actomyosin-mediated contractile force generation in the vasculature, thereby playing a role in smooth muscle contraction, cell motility and adhesion. Recent evidence supports an important role for ROCK in the increased vasoconstriction and remodeling observed in various models of hypertension. This review will provide a commentary on the development of specific ROCK inhibitors and their clinical application. Fasudil will be discussed as an example of bench-to-bedside development of a clinical therapeutic that is used to treat conditions of vascular hypercontractility. Due to the wide spectrum of biological processes regulated by ROCK, many additional clinical indications might also benefit from ROCK inhibition. Apart from the importance of ROCK in smooth muscle contraction, a variety of other protein kinases are known to play similar roles in regulating contractile force. The zipper-interacting protein kinase (ZIPK) and integrin-linked kinase (ILK) are two well-described regulators of contraction. The relative contribution of each kinase to contraction depends on the muscle bed as well as hormonal and neuronal stimulation. Unfortunately, specific inhibitors for ZIPK and ILK are still in the development phase, but the success of fasudil suggests that inhibitors for these other kinases may also have valuable clinical applications. Notably, the directed inhibition of ZIPK with a pseudosubstrate molecule shows unexpected effects on the contractility of gastrointestinal smooth muscle.

## 1. Introduction

Smooth muscle plays an important role in the regulation of vascular tone, bronchial diameter, gastrointestinal motility, penile erection and parturitional/post-parturitional myometrial contraction as well as a myriad of other biological functions. Not surprisingly, contractile abnormalities of smooth muscle are considered to underlie many diseases and disorders, including hypertension, vasospasm, diabetes-associated microvascular abnormalities, bronchial asthma, preterm labor, urinary incontinence, megacolon and irritable bowel syndrome. These abnormalities can involve alterations in spontaneous activity and pace-making, changes in responsiveness to neuronal and/or hormonal influences, development of ultra-structural changes to the muscle layer, morphological perturbations to the contractile machinery and changes in both intra- and inter-cellular signal transmission. Current surgical and interventional therapies, while efficient in certain clinical settings, are costly and primarily palliative, and do not target the cause of the disease. The expanding population of patients in need of treatment is associated with significant cost to health care and society. Thus, attention is being focused more and more on a molecular approach to the treatment of various smooth muscle-associated disorders and diseases. Success in this endeavor requires a detailed understanding of the molecular basis of smooth muscle function and regulation, identification of abnormalities (dysfunctional proteins and signaling pathways) leading to contractile pathologies and development of strategies to reverse such abnormalities. This review will focus on the downstream effector protein kinases that elicit smooth muscle contraction without a concurrent increase in intracellular Ca^2+^ (the Ca^2+^-sensitization phenomenon): Rho-associated kinase (ROCK), zipper-interacting protein kinase (ZIPK) and integrin-linked kinase (ILK). Because these protein kinases are integral to the proper contractile function of smooth muscle, the development of therapeutic modalities has emerged as an attractive pursuit. Other protein kinases (e.g., cAMP-dependent protein kinase (PKA), cGMP-dependent protein kinase (PKG), Ca^2+^/phospholipid-dependent protein kinases (PKC) and various mitogen-activated protein kinases (MAPK)) also play key regulatory roles in the Ca^2+^-independent, smooth muscle contraction. Due to space limitations, an in depth review of these later protein kinases will not be undertaken in this review; the reader is directed to a number excellent and comprehensive reviews of these additional signaling pathways [1,2,3,4].

## 2. Mechanism of Smooth Muscle Contraction

**Figure 1:**
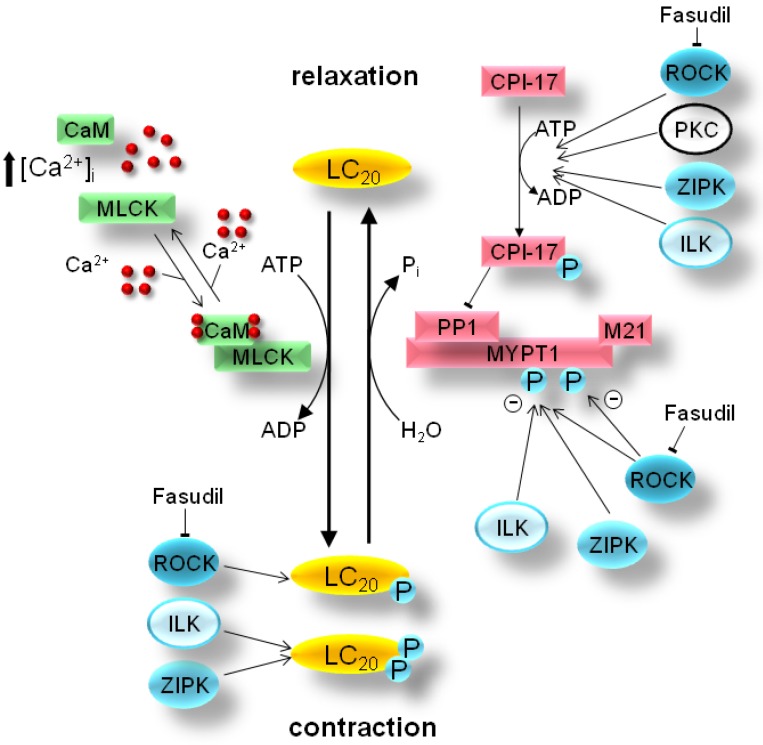
Smooth muscle Ca^2+^ sensitization: Smooth muscle contraction is activated by phosphorylation of the 20-kDa light chains of myosin II (LC_20_) by Ca^2+^/calmodulin (CaM)-dependent myosin light chain kinase (MLCK). Agonist-stimulation of G-protein-coupled receptors results in the activation of various protein kinase pathways (*i.e.*, Rho-associated protein kinase, ROCK; integrin-linked kinase, ILK; and/or zipper-interacting protein kinase, ZIPK) to elicit downstream inhibition of myosin phosphatase (PP1+MYPT1+M21) activity, increased LC_20_ phosphorylation and smooth muscle contraction. Ca^2+^ sensitization can be generated either directly by phosphorylation of MYPT1 (the myosin-targeting subunit of myosin phosphatase) or indirectly via phosphorylation of CPI-17 [PKC (protein kinase C)-potentiated inhibitory protein for PP1 (protein phosphatase type 1) of 17-kDa]. Moreover, Ca^2+^-independent LC_20_ diphosphorylation by ZIPK and/or ILK is revealed during inhibition of myosin phosphatase activity with pharmacological agents such as microcystin or in some smooth muscle pathologies.

Smooth muscle can be broadly classified into the slowly-contracting tonic muscles of the vasculature and the rapidly-contracting phasic muscle of the viscera [1]. While there are many variations to the general theme, as outlined in several recent reviews [1,2,3,4], the general mechanism of smooth muscle contraction is dependent on the control of intracellular, free Ca^2+^ levels (Figure 1). The canonical excitation-contraction coupling pathway is triggered by neural or hormonal stimulation to elicit the influx of extracellular or intracellular (from the sarcoplasmic reticulum) Ca^2+^ into the cytosol. This process is the result of G-protein-coupled receptor activation by a variety of signaling molecules. 

For example, contractile signaling in the gastrointestinal smooth muscle [3] can include acetylcholine stimulation of M_2_ and M_3_ muscarinic receptors, ATP/UTP stimulation of P2Y2 receptors, S1P stimulation of S1P_2_ receptors and NPY/PP stimulation of Y_2_/Y_4_ receptors. In the vascular smooth muscle [5], the contractile signaling can include thromboxane stimulation of G_12/13_-coupled receptors, endothelin stimulation of ET_A_/ET_B_ receptors, norepinephrine stimulation of α_1_-adrenergic receptors, angiotensin II stimulation of AT_1_ receptors and vasopressin stimulation of V_1_ receptors. The receptors are coupled to downstream G-proteins (G_q_ and G_i_) and activate phospholipase C to stimulate phosphoinositide hydrolysis, resulting in the generation of diacylglycerol and inositol 1,4,5-trisphosphate (IP_3_). This diffusible Ca^2+^-mobilizing messenger binds to and opens receptors that release Ca^2+^ from the sarcoplasmic store. Cytoplasmic Ca^2+^ then binds to calmodulin, initiating the activation of myosin light chain kinase (MLCK) and subsequent phosphorylation of the 20-kDa regulatory light chains of myosin II (LC_20_) on Ser-19. The phosphorylation of LC_20_ is associated with an increase in the actin-activated myosin ATPase activity and enables force development via cross-bridge cycling. The contraction is terminated by removal of Ca^2+^ as well as dephosphorylation of LC_20_ by myosin light chain phosphatase (MLCP) [6]. An additional mechanism for smooth muscle contraction is not dependent on Ca^2+^-dependent activation of MLCK, but rather on protein kinases that can phosphorylate LC_20_ directly or alter LC_20_ phosphorylation status by affecting upstream regulators (Figure 1). In this situation, the sustained contractile force generated upon agonist stimulation does not parallel intracellular Ca^2+^ levels [7]. Thus, the force/Ca^2+^ ratio is variable, an effect termed Ca^2+^ sensitization/desensitization [1]. 

A variety of Ca^2+^-independent protein kinases have been implicated in the phosphorylation of LC_20_ on Ser-19 (as discussed in [8,9]), including Rho-associated protein kinase (ROCK), p21-activated protein kinase (γ-PAK), citron kinase, integrin-linked protein kinase (ILK), zipper-interacting protein kinase (ZIPK), MAPK-activated protein kinase 1b (MAPKAPK-1b or RSK-2) and MAPKAPK-2. Although the physiological role of these protein kinases is not clear at present, ILK and ZIPK are the only two kinases found to be responsible for diphosphorylation of LC_20_ in smooth muscle [8,11,12,13]. The diphosphorylation of LC_20_ on both Thr-18 and Ser-19 further increases the actin-activated myosin ATPase activity over that observed with Ser-19 phosphorylation alone [10]. Ca^2+^ sensitization is also elicited through protein kinase-mediated inhibition of myosin light chain phosphatase (MLCP) [1,14]. The inhibition of MLCP prolongs LC_20_ phosphorylation and maintains the smooth muscle in a contracted state. MLCP is a type 1 protein serine/threonine phosphatase that exists as a heterotrimer, composed of a 37-kDa catalytic PP1c subunit, a 110-kDa to 130-kDa myosin targeting MYPT1 subunit and a 20-kDa M20 subunit [6]. Protein kinase-mediated attenuation of MLCP activity can occur with the phosphorylation of the MYPT1 subunit at either of two inhibitory phosphorylation sites, Thr-697 or Thr-850. Alternatively, MLCP activity can be attenuated indirectly via the phosphorylation of CPI-17 [the PKC (protein kinase C)-potentiated inhibitory protein for PP1c of 17-kDa] that increases the inhibitory potential of CPI-17 for MLCP.

The phenomenon of Ca^2+^ desensitization also has important implications for the contractile state of smooth muscle [1,3]. The release of cyclic nucleotides (cGMP and/or cAMP) in response to hormonal or neuronal stimulation promotes smooth muscle relaxation either by lowering intracellular [Ca^2+^] or desensitizing the muscle to Ca^2+^. Nitric oxide stimulates cGMP production by guanylyl cyclase and activation of PKG. This kinase can lower [Ca^2+^] through multiple mechanisms, but it also raises the Ca^2+^ threshold for contraction, thus causing Ca^2+^ desensitization. The mechanism of Ca^2+^ desensitization has been suggested to involve the direct activation of MLCP by PKG (and/or PKA). Later studies identified the major sites phosphorylated on MYPT1 by PKA and PKG [97] and found that phosphorylation of Ser-696 by PKG could prevent the subsequent phosphorylation of Thr-697 by ZIPK and the inhibition of MLCP activity [97,98]. 

## 3. Pathology of Smooth Muscle Contraction

The coordinated regulation of contraction is a key property of smooth muscle, which when functioning normally, contributes to general health and wellness, but when dysfunctional is associated with morbidity and mortality. Ca^2+^ sensitization exerts fine control of smooth muscle tone which is essential for maintenance of normal tissue and organ function. Pathological alterations in the sensitivity of smooth muscle tissues to Ca^2+^ have been hypothesized to underlie many diseases/disorders associated with smooth muscle dysfunction, including hypertension, vasospasm, urinary incontinence, asthma, preterm labor and intestinal dysmotility. 

While monophosphorylation of LC_20_ at Ser-19 is observed in smooth muscle tissues in response to physiological contractile stimuli, the activation of Ca^2+^-independent protein kinases is integral to the development of LC_20_ diphosphorylation and sustained contractile force commonly observed in pathological conditions. For example, the increased vascular constriction observed after PGF_2__α_ treatment of hyperplastic rabbit carotid artery correlated with higher levels of diphosphorylated LC_20 _[15]. Cerebral vasospasm is a serious, often fatal, complication of subarachnoid hemorrhage, and during vasospasm in the canine cerebral artery, levels of LC_20_ diphosphorylation were increased over control [16,17]. Furthermore, LC_20_ diphosphorylation was significantly elevated in vessels isolated from a porcine model of cerebral artery vasospasm [18]. 

Altered smooth muscle contractility has also been observed in gastrointestinal disorders (e.g., Crohn`s disease, ulcerative colitis, Hirschsprung’s disease and toxic megacolon) [19,20,21]. With intestinal inflammation, it is thought that smooth muscle cells undergo a phenotypic change whereby normal rhythmic contractions are supplanted by sustained Ca^2+^-independent contractions that persist long after the mucosal response to injury has subsided. In overt inflammatory conditions of the bowel, such as Crohn’s disease and ulcerative colitis (*i.e.*, Inflammatory Bowel Disease, IBD), there have been longstanding observations of altered motility and impaired function of the intestinal smooth muscle. Smooth muscle from the inflamed intestine of Crohn’s disease patients [22] or ulcerative colitis patients [23] exhibited increased contractility following stimulation with contractile agonist. On the other hand, interleukin-1β, a proinflammatory cytokine that plays a role in IBD, was increased in colonic circular muscle of patients with ulcerative colitis and believed to contribute to smooth muscle hypocontractility [21]. So, contractile dysfunction in IBD appears to depend on the intestinal region and the inflammatory stimulus.

Smooth muscle beds display diverse contractile phenotypes and possess a variety of functional signaling mechanisms to regulate force development. These differences should enable the use of an appropriate pharmacological agent to affect the contractile tone of specific tissues. Three protein kinases have emerged as the best studied and perhaps most important contributors to smooth muscle Ca^2+^-sensitization: ROCK, ILK and ZIPK. These protein kinases have been proven to alter smooth muscle contractility through diphosphorylation of LC_20_ or inhibition of MLCP through phosphorylation of CPI-17 and/or MYPT1.

## 4. Bench-to-Bedside Success: The Story of the ROCK-Inhibitor Fasudil

**Figure 2:**
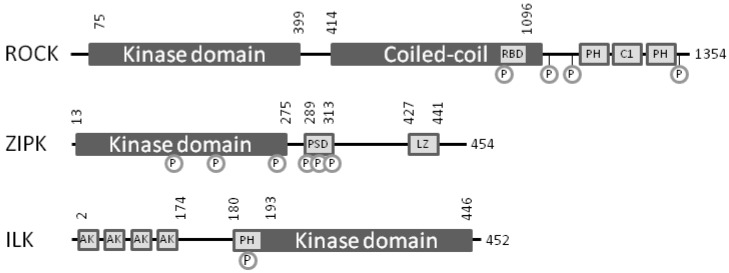
Catalytic and regulatory domains of the smooth muscle Ca^2+^-sensitizing protein kinases ROCK, ILK and ZIPK. The domains of human ROCK, ZIPK and ILK are identified by the starting and ending residues. Regulatory phosphorylation sites reported in the literature have been marked. Abbreviations: For ROCK: RBD, Rho-binding domain; PH, pleckstrin homology domain; C1, protein kinase C conserved region. For ZIPK: PSD, pseudosubstrate domain; LZ, leucine zipper domain. For ILK: AK, ankyrin repeat; PH, pleckstrin homology domain.

There are two isoforms in the ROCK kinase family (ROCK-1 & ROCK-2), activated by binding to the small G-protein RhoA-GTP, downstream of G-protein-coupled receptors. ROCK possesses an amino-terminal Ser/Thr kinase domain, a Rho-binding domain (RBD), a coiled-coil region and carboxyl-terminal pleckstrin-homology domain (PHD) and cysteine-rich domain (CRD) [24,25] (Figure 2). Binding of RhoA-GTP to the RBD situated within the coiled-coil region relieves the autoinhibitory interaction between the C-terminus and the kinase domain, thereby activating ROCK. Proteolytic cleavage of ROCK by caspases or granzyme B can generate a constitutively active kinase through the removal of the autoinhibitory region. Although less understood, some studies report that ROCK activity can also be modulated by phosphorylation of residues within the carboxyl-terminal region [26]. Additional phosphorylation-dependent mechanisms of ROCK regulation are likely to be identified. As a regulator of smooth muscle contraction, ROCK was the first protein kinase found to promote Ca^2+^ sensitization by phosphorylation of MYPT1 at the two major inhibitory sites [27,28]. In addition to phosphorylating MYPT1 at the inhibitory Thr-697 and Thr-850 residues, ROCK also has been shown to mediate the phosphorylation of LC_20_ at Ser-19 [29] and CPI-17 at Thr-38 [30].

**Figure 3:**
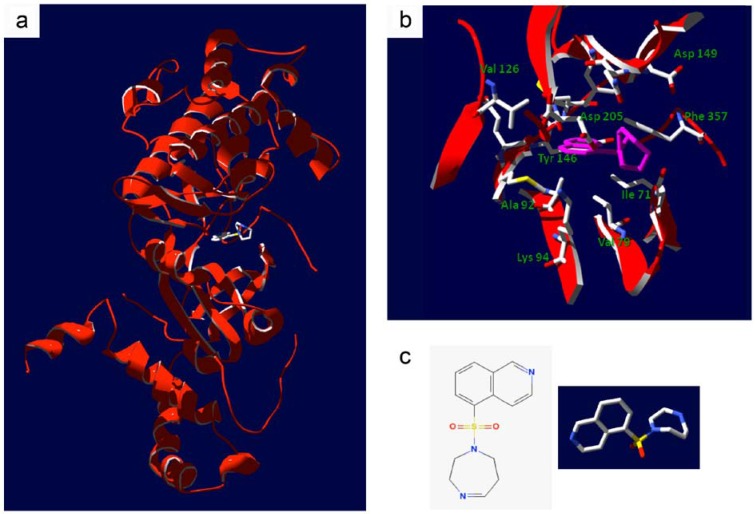
Structure of the ROCK kinase domain in complex with fasudil. **(a)**, Ribbon diagram detailing the crystal structure of the bovine ROCK kinase domain in complex with fasudil, (residues 18 to 417, PDB ID: 2F2U [34]). **(b)**, Detail of fasudil occupancy within the ATP binding pocket of ROCK; amino acid side chains lining the pocket within 6 Å of fasudil are shown. Fasudil is shown in pink. **(c)**, The molecular structure of fasudil in 2- and 3-dimensional renderings.

Many recent reviews document the role of ROCK in a variety of diseases and its suitability as a therapeutic target [24,31,32]. Examples of relevant pathophysiologies with a defined ROCK linkage are hypertension, cerebral and coronary vasospasm, bronchial asthma, preterm labor, atherosclerosis and many aspects of cancer (e.g., oncogenic transformation, neoangiogenesis, cell motility, migration and metastasis). However, a limitation of many studies carried out on ROCK is the low specificity of pharmacological inhibitors that fail to distinguish ROCK-1 from ROCK-2 as well as other protein kinases [33]. To date, the most promising ROCK inhibitor is the isoquinolinesulfonamide, fasudil (HA-1077, Figure 3), which was first described as a Ca^2+^ antagonist with the ability to relax canine basilar artery following contraction with a calcium ionophore [35]. 

Fasudil could lower the effect of delayed cerebral vasospasm from subarachnoid hemorrhage (SAH) that may occur after an episode of bleeding into the subarachnoid space following head trauma or cerebral aneurysm rupture. SAH triggers blood vessel spasm that dramatically lowers blood flow, leading to tissue damage and/or stroke. The success and safety of fasudil in the treatment of SAH promise that it might be of use in a variety of indications. Fasudil has been in clinical use for SAH in Japan since 1995 and is currently in clinical testing for additional indications, reflecting the importance of ROCK activity in a variety of cardiovascular diseases. A phase II study is investigating the ability of fasudil to increase endothelium-dependent relaxation in coronary artery disease, and a phase III study concerning its use in Raynaud's phenomenon and scleroderma is also underway. Raynaud's phenomenon is thought to occur from vasospasm of the digital arteries and cutaneous arterioles, causing atrophy. A phase II study also found that fasudil could improve chronic stable angina by increasing the ischemic threshold of patients [36]. Reflecting a novel connection between ROCK and learning, fasudil was demonstrated to improve spatial learning and working memory in rats and may be of use in the treatment of dementia and Alzheimer’s disease [32]. Other indications positively influenced by fasudil are acute ischemic stroke [37], atherosclerosis [38,39], aortic stiffness [40], cerebral blood flow [41], coronary artery spasm [42,43], cardiac and cerebral aneurysm [44,45], glaucoma due to hypertension [46], heart failure-associated vascular resistance and constriction [47] as well as hypertension [48,49]. The latter two reports showed that inhibition of ROCK relaxes vessels, as measured by an increase of blood flow and a concomitant drop of increased blood pressure. These studies established fasudil as a possible anti-hypertensive drug which is of importance given the significant off-target effects of current treatments. 

Interestingly, fasudil was not developed as a ROCK inhibitor *per se*, but as a drug to relax blood vessels, and initially, it was unclear what protein was actually targeted in the vasculature by the drug. Originally, fasudil was described to inhibit PKA and PKC, but later it was found that the drug was a much more potent inhibitor of ROCK [50]. It is still unclear if off-target effects of fasudil contribute to its clinical success. For example, a recent report provides evidence that small molecule inhibitors of ROCK can act on divergent kinome family members [51] with similar potency as ROCK. New ROCK inhibitors are being tested; for example, SAR407899 is approximately eight-times more potent than fasudil (K_i_ values: 36 nM for SAR407899 *vs.* 271 nM for fasudil [52]) and both SAR407899 and SB-772077-B can lower blood pressure in rats [53]. Based on the relative importance of ROCK, ZIPK and ILK in the regulation of smooth muscle contraction [8,54,55], selective inhibitors to the latter two protein kinases might also have important clinical applications.

## 5. Zipper-Interacting Protein Kinase

Zipper-interacting protein kinase ((ZIPK), also known as DAPK3 or Dlk) [56] belongs to the family of death-associated protein kinases (DAPK) [57,58]. ZIPK controls a variety of cell processes, including cell motility [59] and smooth muscle contraction [12,60,61]. Identified in 1998 [62,63], ZIPK possesses an amino-terminal kinase domain, a putative central autoinhibitory domain and a carboxyl-terminal leucine zipper motif that permits dimerization and interactions with other proteins (Figure 2). As a regulator of cellular motility, ZIPK can phosphorylate non-smooth muscle myosin light chains [59] to cause re-organization of the actin cytoskeleton. ZIPK could direct LC_20_ phosphorylation and was necessary for cell motile processes in mammalian fibroblasts [59]. In smooth muscle, ZIPK is associated with MLCP [61,64] and inhibits its activity by phosphorylation of MYPT1 at Thr-697 [60,61]. In addition, ZIPK can drive Ca^2+^-independent diphosphorylation of LC_20_ at both Thr-18 and Ser-19 [11,12,13,60], and ZIPK may regulate MLCP activity indirectly since it is able to phosphorylate CPI-17 *in vitro* [65]. These findings provide good evidence that ZIPK plays a key role in the regulation of smooth muscle contraction. Indeed, early reports described ZIPK as the main kinase responsible for Ca^2+^-independent contraction in vascular smooth muscle [12,64]. Additional Ca^2+^-sensitizing protein kinases such as integrin-linked kinase (ILK), protein kinase C (PKC) and ROCK are also found in vascular smooth muscle beds, and the relative importance of each kinase pathway remains to be elucidated. Since ZIPK is expressed in various non-vascular smooth muscle tissues such as bladder and intestine [66,67], the exact effect of systemic inhibition of ZIPK cannot be predicted.

The kinase domain of ZIPK is most similar to other DAPKs (e.g., DAPK1) but also shares significant sequence and structural conservation with MLCK [57]. The activities of DAPK1 and MLCK are controlled by intracellular Ca^2+^. The binding of Ca^2+^-calmodulin removes an autoinhibitory, pseudosubstrate domain and regulates their kinase activities. The autoinhibitory domains of DAPK1 and MLCK act as pseudosubstrates since they share sequence similarity with their substrate target phosphorylation sites. In addition, these domains are subject to phosphorylation (Ser-308 in DAPK1 [69,70] & Ser-815 in MLCK [71]) that increases pseudosubstrate binding to the active site, thereby increasing the concentration of Ca^2+^-calmodulin necessary for half-maximal activation and reducing kinase activity. ZIPK is distinguished from the DAPKs and MLCK since it lacks a calmodulin-binding domain. Thus, its activity is regulated independently of Ca^2+^-calmodulin; however, its activity can be regulated by phosphorylation *in vivo* and *in vitro* [70,71,72,73,74,75]. Three (Thr-299, Thr-309 and Ser-311) of ZIPK’s six phosphorylation sites are located within a region that has similarity with the autoinhibitory domain of MLCK and DAPK [74]. Mutation of these phosphorylation sites to alanine moderately enhanced ZIPK activity towards LC_20_ and MYPT1 *in vitro* as well as increased cell detachment *in vivo*, a sign of increased ZIPK activity [74]. In addition, truncation of ZIPK to eliminate this domain greatly increased ZIPK activity [74]. This suggests that ZIPK regulation by an autoinhibitory domain and phosphorylation within or close to this domain reduces ZIPK activity, similar to the regulation observed for DAPK1. Recent evidence also supports a role for ubiquitination in the regulation of ZIPK [76] as the ubiquitin ligase UbcH5c is able to influence ZIPK protein accumulation. The main ubiquitination sites have been putatively mapped to the N-terminal kinase domain of ZIPK, and their precise identification may help clarify the contribution of this regulatory mechanism to ZIPK’s role in smooth muscle pathophysiology. 

**Figure 4:**
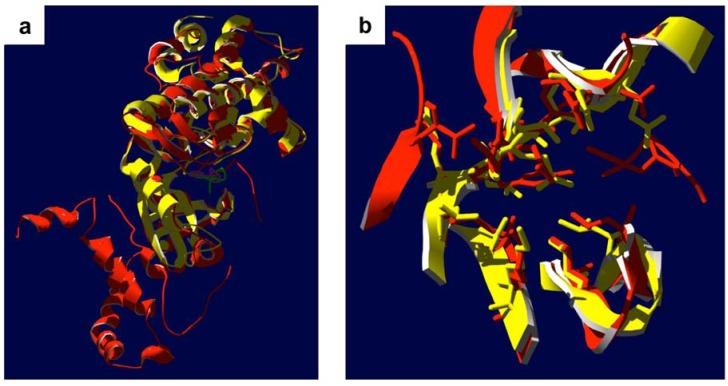
Structural comparison of ZIPK and ROCK. **(a)**, Overlaid ribbon diagrams of the crystal structures of the ROCK kinase domain (red, PDB ID: 2F2U; residues 18–417 [34]) in complex with fasudil (pink) and the ZIPK kinase domain (yellow, PDB ID: 3BHY, residues 9–289 [68]) in complex with the inhibitor (4r)-7,8-dichloro-1',9-dimethyl-1-oxo-1,2,4,9-tetrahydrospiro-[beta-carboline-3,4'-piperidine]-4-carbonitrile (green). **(b)**, Detail of ATP binding pockets of aligned ROCK and ZIPK structures; amino acid side chains lining the pockets within 6 Å of the inhibitors are shown. For clarity, the inhibitor structures were omitted.

Biochemical analyses performed *in vitro* suggest that fasudil and other ROCK selective inhibitors do not influence the activity of ZIPK [13,61]. A structural alignment of the ATP-binding pockets of ROCK and ZIPK illustrates the possible molecular determinants for the targeting specificity of fasudil and related ROCK inhibitors (Figure 4). Two prominent residues within the ATP-binding pocket of ROCK are observed to make molecular contact with fasudil (Figure 3). The Tyr-146 residue of ROCK packs against the heterocyclic isoquinoline element of fasudil; furthermore, the Phe-357 residue of ROCK appears to interact with fasudil’s diazepane ring. The two identified residues are not conserved in the ZIPK sequence, with replacement of the corresponding residues to non-aromatic side-chains (*i.e.*, amino acids Tyr-146 and Phe-357 of ROCK for Leu-95 and Ala-334 of ZIPK, respectively). The consequence of the Tyr-146 amino acid replacement is illustrated in the structural alignment of ZIPK and ROCK provided in Figure 4. Unfortunately, the available ZIPK structures do not include the Ala-334 residue, so it is unknown how this region of ZIPK contributes to its structural topology. Still, the space available within the ZIPK ATP-binding pocket for inhibitor occupancy is dramatically different from that of ROCK, especially given the absence of an aromatic residue corresponding to Tyr-146.

Specific small molecule inhibitors for ZIPK have not been readily available for application to studies of smooth muscle biology; however, Okamoto and colleagues have recently identified novel potent and selective DAPK inhibitors with structure-based virtual screening [77,78]. As the nearest relatives, DAPK1 and DAPK2 show ~80% sequence conservation with ZIPK (DAPK3). Moreover, an alignment of the kinase domains of ZIPK (PDB ID: 3BHY) and DAPK1 (PDB ID: 1P4F) demonstrates that the structural orientation of each is nearly identical.

In their study [78], Okamoto and colleagues identified six compounds with a 2-phenyl-4-(3-pyridinylmethylene)-5(4*H*)-oxazolone core structure to have excellent selectivity for DAPKs. Each compound inhibited DAPK1 and ZIPK (DAPK3) with IC_50_’s in the low micromolar range without inhibition of the other Ser/Thr- or Tyr-kinases tested. Although the selectivity of the compounds have yet to be tested against other important smooth muscle contractile protein kinases (*i.e.*, ROCK, ILK & MLCK), the DAPK inhibitors may have potential for development of pharmacological treatments for smooth muscle contractile disorders involving ZIPK. 

Synthetic oligopeptides based on the pseudosubstrate autoinhibitory domains may offer alternatives to small molecule inhibitors of the ATP-binding pocket in the kinase domain [79]. Traditionally, pseudosubstrate peptides are considered less attractive inhibitors when compared with small molecule drugs due to several limitations, including delivery and stability problems. However, progress has been made to reduce the severity of these limitations [79,80]. As outlined above, ZIPK appears to be maintained in an inhibited state by a pseudosubstrate autoinhibitory domain [66,74]. This intrasteric regulation [81] can be overcome by synthetic peptides containing a similar number and spatial arrangement of residues found to be important for substrate recognition. The peptide substrate antagonists SM1 and AV25, derived from the autoinhibitory domain sequence of MLCK, have been used to inhibit ZIPK activity [66,67]. Bacterially-expressed ZIPK (kinase domain only: amino acids 1–320) as well as myc-tagged, full-length ZIPK immunopurified from human cells, were both inhibited by the two peptide antagonists *in vitro* [57,66]. In agreement with the suggestion that the peptide antagonists are similar to the autoinhibitory domain of ZIPK, the nature of the inhibition was competitive. In rat ileum, the SM1 peptide antagonist inhibited LC_20_ diphosphorylation and contractile force induced by addition of exogenous ZIPK [66]. In contrast, the AV25 peptide had no effect on Ca^2+^-independent LC_20_ phosphorylation in rat caudal artery smooth muscle, suggesting that either endogenous ZIPK was not responsible or that ZIPK was not targeted by AV25 in this tissue [8]. Further studies in rat ileal and colonic smooth muscle showed increased contraction (rather than the expected decrease) following the addition of AV25 [67], but not SM1. As expected, the observed sensitization of contractile force with AV25 treatment was accompanied by increases in LC_20_ diphosphorylation and MYPT1 phosphorylation. The difference in contractile effects observed *in situ* with AV25 and SM1 pseudosubstrate peptides is puzzling. It is conceivable that SM1 is phosphorylated *in situ,* and this event is important for inhibitory function within the muscle tissue. Experiments should be performed to examine the contractile effects of the SM1 peptide following its thiophosphorylation. While the specific mechanism whereby AV25 augments Ca^2+^ sensitization in gastrointestinal smooth muscle remains to be elucidated, AV25 or molecules based on its structure may hold promise in therapeutic applications to induce contractility in conditions of gastrointestinal hypomotility.

## 6. Integrin-Linked Protein Kinase

Integrin-linked protein kinase (ILK) was originally identified as a binding partner for the integrin β cytoplasmic tail [82] and was demonstrated to play a key role in coupling transmembrane integrin receptors with the actin cytoskeleton. The molecular structure of ILK can be divided into three domains: (1) an ankyrin repeat domain located in the amino-terminus is necessary for association with adaptor proteins and localization of ILK to focal adhesions, (2) a central pleckstrin-homology domain (PHD) mediates binding with phosphatidylinositol (3,4,5)-trisphosphate (PIP_3_), and (3) a carboxy-terminal kinase domain that is capable of phosphorylating a diverse array of substrate proteins (Figure 2). The ILK kinase domain is also able to mediate interactions with integrins and paxillin as well as the parvins, thereby connecting ILK to the actin cytoskeleton. ILK is highly expressed in cardiac muscle, but also plays a role in smooth muscle contraction, vascular development, tumor angiogenesis and many aspects of cancer [83,84,85,86]. The function of ILK seems to be dual: firstly, it acts as a scaffolding protein, linking integrins to the actin cytoskeleton and regulating actin polymerization; secondly, it acts as a Ser/Thr-protein kinase. 

**Figure 5:**
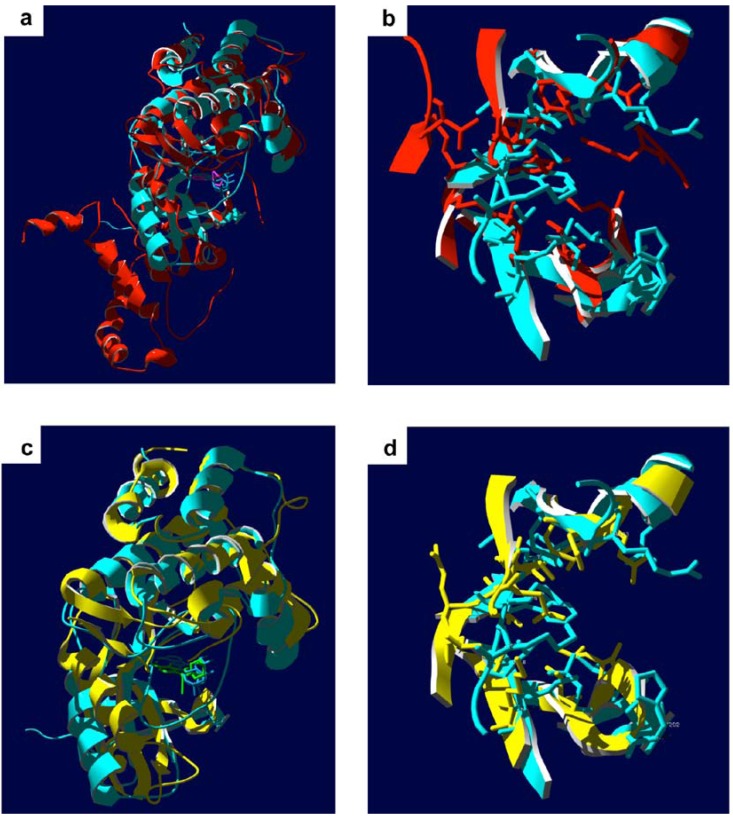
Structural comparison of ILK with ZIPK and ROCK. **(a)**, Overlay of the crystal structures of the ILK kinase domain (aquamarine; PDB ID: 3KMW; residues 183–452 [89]) in complex with ATP (blue) and the ROCK kinase domain (red; PDB ID: 2F2U; residues 18–417 [34]) in complex with fasudil (pink). **(b)**, Detail of ATP binding pockets of aligned ILK and ROCK structures; amino acid side chains lining the pockets within 6 Å of the ATP and fasudil molecules are shown. For clarity, the ATP and fasudil structures were omitted. **(c)**, Overlay of the crystal structures of the ILK kinase domain (aquamarine; PDB ID: 3KMW) in complex with ATP (blue) and the ZIPK kinase domain (yellow; PDB ID: 3BHY; residues 9 to 289; [68]) in complex with its inhibitor (green) as described in Figure 4b. **(d)**, Detail of ATP binding pockets of aligned ILK and ZIPK structures; amino acid side chains lining the pockets within 6 Å of the ATP and inhibitor molecules are shown. For clarity, the ATP and inhibitor structures were omitted. Note that ILK structure was solved in complex with the inhibitory protein α-parvin [89], which is not shown.

There has been considerable discussion regarding the kinase activity of ILK. Genetic analyses have suggested that the protein kinase domain may not be required for ILK’s involvement in biological processes since deletion of this domain had no detectable effect [84,87]. Moreover, ILK lacks residues of its protein kinase domain thought to be necessary for catalytic function (*i.e.*, phosphoryl transferase activity) [88]. The recent co-crystal structure of the ILK kinase domain with the inhibitor α-parvin sought to resolve this discussion [89]. The structural analysis revealed a distinct inactive kinase domain in ILK, and the authors defined the protein as a pseudokinase. The ILK pseudokinase domain possesses a dramatically altered ATP-binding loop (P loop) where the canonical glycine-rich GXGXXG motif found in Ser/Thr-kinases is replaced by a non-glycine-rich NENHSG sequence. While maintaining a canonical kinase fold, this pseudoactive ILK kinase domain was proposed to promote the effective assembly of macromolecular complexes rather than performing the catalytic phosphoryl transferase function typically associated with protein kinases [89]. One limitation of this structural study was the co-crystalization of ILK in the presence of the inhibitor protein α-parvin. From this structure, the authors reported significant distortion of the kinase domain structure (Figure 5), an observation that was interpreted to result in the loss of phosphoryl transferase ability. It is difficult to interpret mechanistic aspects of the structure and function of ILK from this single report. The structural distortion of the kinase domain could result from the inactive status of ILK since the protein is in complex with an inhibitor. Although the sequence/structure is unusual for a Ser/Thr-protein kinase family member, a multitude of other research suggests that ILK possesses *bona fide* phosphoryl transferase activity [83,90,91]. Regardless of the controversy surrounding the kinase activity of ILK, the protein stands as an important regulator of smooth muscle contractile function, especially given its dual roles in the assembly of signaling complexes to regulate cytoskeletal structure and/or in Ca^2+^ sensitization via the phosphorylation of target proteins. 

Several studies have examined the role of ILK in the generation of Ca^2+^ sensitization of smooth muscle contraction. Initial investigations detailing this novel role for ILK demonstrated that G-protein-coupled signaling pathways unmask the activity of myofilament-associated ILK to enable the phosphorylation of LC_20_ and trigger contraction [83]. Intriguingly, subcellular fractionation revealed two distinct pools of ILK; one with the expected properties of integrin/focal adhesion-associated ILK and another that was associated with the myofilament. This observation and further investigations provide strong confirmation that the role of ILK as both adaptor protein and kinase is important for smooth muscle contractility. Additional investigations have found that ILK can phosphorylate MYPT1 [92] and CPI-17 [93] to inhibit myosin phosphatase activity in vascular smooth muscle. ILK has been suggested to be responsible for Ca^2+^-independent contraction of a phasic smooth muscle, the circular smooth muscle of the esophagus [55], where it may be involved in a PKCε-activated, ERK1/2 contractile pathway. ILK expression levels also appear higher in intestinal smooth muscle beds when compared with vascular beds [67]. Different expression levels of ILK in these muscle types may also suggest a critical role for the kinase in Ca^2+^ sensitization and the particular contractile properties of these tissues. 

ILK has demonstrated unusual resistance to numerous broad-spectrum kinase inhibitors; yet with its unusual ATP-binding site structure, it should be feasible to design a distinctive inhibitor with specific ILK-targeting potential. Indeed, a few ILK inhibitors have recently emerged from the pharmaceutical pipeline. The compounds were identified in high-throughput screening of a rationally-designed small molecules library and possess approximately 100- to 1000-fold selectivity for ILK over a number of additional kinases tested under similar conditions. The inhibitors KP-SD-1/KP-392 and KP-SD-2 show selectivity for ILK with IC_50_ values of 0.3 μM and 0.8 μM, respectively [91,94]. The inhibitory characteristics of the two molecules were also tested for a variety of other kinases (e.g., PKB, ERK, GSK-3β & PKA), and the IC_50_ values were above 25 μM. Notably, newer derivatives with improved cell permeability, QLT0267 [95] and QLT0254 [90], also inhibit the kinase activity of ILK in cell-free assays. To date, it is not clear whether these compounds also possess inhibitory potential for ZIPK and ROCK, so their usefulness for targeting ILK-dependent contractile disorders may be limited. The structural comparisons of the ATP-binding pockets of ILK, ZIPK and ROCK suggest that some degree of specificity will be possible (Figure 5). Furthermore, examination of the amino acid sequence suggests that the ILK active site is unique when compared to ROCK and ZIPK. A sequence alignment completed for the kinase domain of the three proteins revealed a 36% conservation of sequence between ILK and ROCK or ZIPK. Interestingly, a 47% conservation of sequence was observed between ROCK and ZIPK. With such disparate sequence within the active sites of these kinases, it is unlikely that the alignment data will be useful in the development of inhibitors. However, the structural distortion of the ILK protein kinase domain that is induced by protein binding [89] might be exploited for rational design of unique ILK inhibitors to selectively target different subcellular pools of the kinase. Recalling that two distinct pools of ILK exist in smooth muscle cells (*i.e.*, one pool with integrin/focal adhesion association and another with myofilament association) [83], it would be beneficial to provide selective inhibition of only the contractile pool of ILK. In this regard, it could be expected that the binding of ILK with different intracellular proteins may elicit variations in the molecular topology of the ATP-binding pocket to provide a unique pharmacological target. 

## 7. Conclusions and Outlook

The protein kinases involved in Ca^2+^ independent smooth muscle contraction are useful therapeutic targets in a multitude of diseases. The success of the ROCK inhibitor fasudil demonstrates that a suite of selective inhibitors for smooth muscle protein kinases would particularly advantageous in the clinical arena. However, our understanding of the protein kinases involved in Ca^2+^ sensitization of the different smooth muscle beds is still incomplete. With ZIPK, ILK and ROCK expression identified in all smooth muscle beds examined to date, it has been difficult to describe a distinct contribution of each kinase to smooth muscle contractility. Selective, smooth muscle-targeted knock-out mouse models would appear to be beneficial for the understanding of the *in vivo* function of each protein kinase in Ca^2+^ sensitization. However, the interpretation of knock-out phenotypes could be confounding due to functional redundancies and/or the potential for alterations in signaling constancy that accompany genetic manipulation to the levels of important signaling proteins. For example, knock-out of ZIPK may impart unintended disruptions on leucine zipper-dependent functional interactions among the proteins associated with the contractile filament irrespective of the protein kinase activity. It is an optimistic conclusion that the ILK and ZIPK signaling modules described in this review will evolve into therapeutic targets in smooth muscle disease/disorders. If a functional redundancy among these signaling molecules does indeed exist within smooth muscle beds, then it may prove difficult to selectively target a particular kinase within a selective smooth muscle bed in the clinical setting. Also, the broad expression of ZIPK and ILK in different cell types may imply numerous side-effects in addition to the desirable reduction of Ca^2+^ sensitivity in smooth muscle cells. Nevertheless, the validation of specific inhibitors (or activators) [96] for either the ILK or ZIPK pathway would be useful in promoting our basic understanding of these signaling pathways in smooth muscle. Pharmacological agents that act to selectively modulate protein kinase activities could be rapidly applied and would be predicted to have minimal effects on the expression levels of other signaling molecules. In the case of ILK, its unusual pseudokinase domain structure makes it a particular interesting pharmacological target.
